# Head and Neck Cancer with Dermatomyositis: A Report of Two Clinical Cases

**DOI:** 10.1155/2010/401825

**Published:** 2010-05-31

**Authors:** Kohichi Yamauchi, Yasunao Kogashiwa, Hiroshi Nagafuji, Naoyuki Kohno

**Affiliations:** Department of Otolaryngology, Head and Neck Surgery, Kyorin University School of Medicine, 6-20-2 Shinkawa, Mitaka, Tokyo 181-8611, Japan

## Abstract

Dermatomyositis is well known to be associated with several types of malignancy and patients with dermatomyositis have higher rates of mortality from cancer. Although rare in Japan, head and neck cancer, especially nasopharyngeal cancer, is the predominant type of cancer associated with dermatomyositis in several areas in Asia, including Hong Kong and Singapore. Here we report two cases of head and neck cancer with dermatomyositis as well as a literature review. Both cases were treated with chemotherapy and radiotherapy. Although the patients were immunosuppressed due to dermatomyositis treatment, no grade 3 or 4 adverse events occurred.

## 1. Introduction

Dermatomyositis is a connective tissue disease with characteristic cutaneous manifestations and it is well known that dermatomyositis is associated with various types of cancer. The first suggestion that dermatomyositis was associated with cancer was made by Sterz in 1916 [1] and numerous cases have since been reported. In this paper, two cases of patients with head and neck cancer along with dermatomyositis are reported.

## 2. Case Reports

### 2.1. Case 1

**Figure 1:**
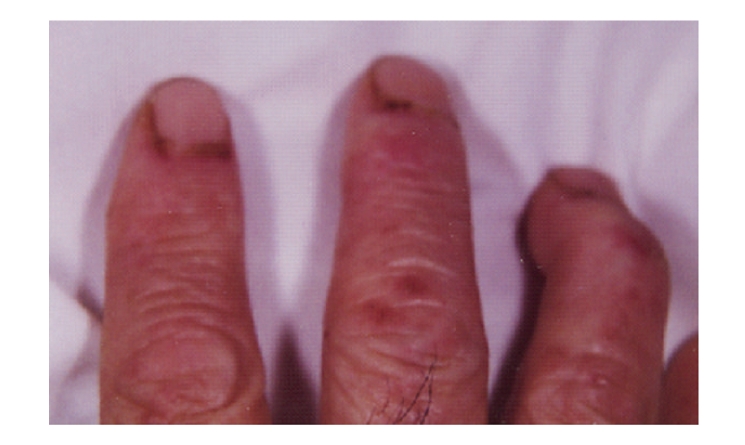
Gottron's sing on the dorsal surface of the proximal interphalangeal joint. Erythematous areas at the bases of the fingernails are seen.

**Figure 2:**
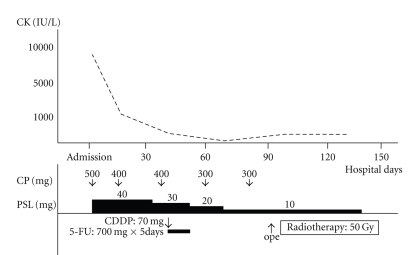
Case 1: Clinical Course. Treatment and serum CK levels are shown. CP: cyclophosphamide.

A 79-year-old man was admitted to our hospital with nasal bleeding and left cheek swelling suggestive of maxillary sinus carcinoma. A solid mass with aggressive bone destruction was detected in the left maxillary sinus on contrast-enhanced computed tomography images. For 1 month, the patient had noticed a systemic rash, the typical heliotrope coloration of the upper eyelids, Gottron's sign on the dorsal surface of his proximal interphalangeal joints, erythematous areas at the bases of the fingernails (Figure 1), dysphagia, and proximal muscle weakness of both extremities, but he and his family did not think that the symptoms were serious. On the second day of hospitalization, his respiratory dysfunction progressed rapidly. His chest X-ray was normal and initial investigations revealed a normal complete blood count and negative antinuclear antibody and anti Jo-1 antibody levels. However, there were several abnormal laboratory test results, including increases in aspartate aminotransferase (AST) (451 IU/L; normal range, 8–33 IU/L), alanine aminotransferase (ALT) (90 IU/L; normal range, 3–30 IU/L), creatine kinase (CK) (8,800 IU/L; normal range, 15–166 IU/L), lactate dehydrogenase (LDH) (973 IU/L; normal range, 118–226 IU/L), and aldolase (ALD) (23.3 IU/L; normal range, 0.60–7.60 IU/L). His clinical symptoms fulfilled the criteria for the diagnosis of dermatomyositis and it was thought that his respiratory dysfunction was caused by acute interstitial pneumonia associated with dermatomyositis. Therefore, the patient was immediately treated with 40 mg/day prednisolone, as well as intravenous cyclophosphamide pulse therapy. The patient's clinical course is shown in Figure 2. His dyspnea and dysphagia recovered rapidly after the second cyclophosphamide pulse treatment. Gradually, the patient's other symptoms improved and tapering of the prednisolone treatment was begun. After the patient's general condition had stabilized, investigation of the patient's maxillary sinus tumor was begun. A transgingival biopsy specimen taken from the maxillary sinus showed an undifferentiated carcinoma. The clinical TNM stage was T4N0M0. The patient was first treated with neoadjuvant chemotherapy consisting of a 5-day continuous infusion of 5-fluorouracil (700 mg/day) with an intra-arterial injection of cisplatin (70 mg/body) through the left superficial temporal artery. One month later, the patient underwent partial maxillectomy under general anesthesia; the prednisolone dose was only 10 mg/day at that time. Then, radiotherapy of 50 Gy by Linac was administered to the primary site and the upper neck. The skin exposed to the radiation developed mild moist desquamation, but no exaggerated radiation reactions were noted. No grade 3 or 4 adverse events occurred during treatment. One month after the end of radiotherapy, computed tomography scan and fiberscopic examination revealed no residual cancer in the maxillary sinus.

### 2.2. Case 2

**Figure 3:**
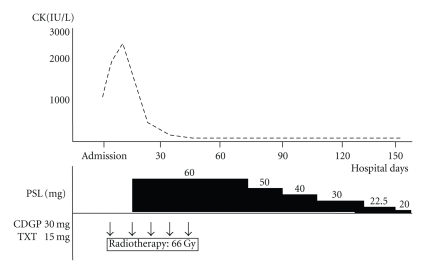
Case 2: Clinical Course. Treatment and serum CK levels are shown.

**Figure 4:**
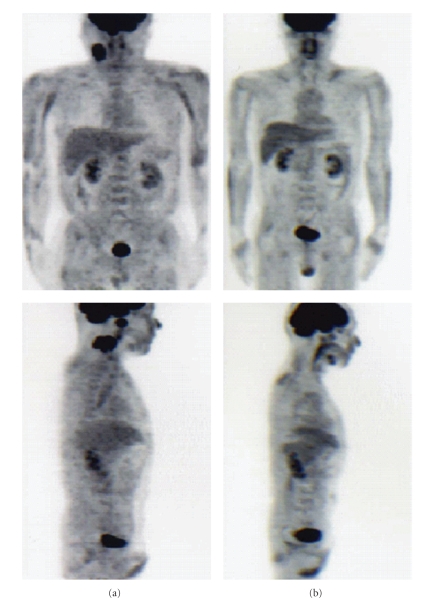
PET-CT. (a) Pre-treatment. Intense FDG uptake is detected in the left cervical region and the nasopharynx. Mild FDG uptake is detected in the muscles of the entire body, especially the upper extremities. (b) Post-treatment. FDG uptake with residual tumor is not detected. FDG uptake in the muscles is improved compared to pre-treatment.

A 42-year-old man was admitted to our hospital for nasopharyngeal carcinoma. The biopsy specimen from the nasopharynx disclosed nonkeratinizing cell carcinoma. The clinical TNM stage was T1N2bM0. On admission, the patient reported general fatigue and dysphagia. A diagnosis of dermatomyositis was made on the basis of a systemic rash, characteristic muscle weakness, compatible skin histological result, and increased levels of CK (1490 IU/L), AST (108 IU/L), ALT (74 IU/L), LDH (447 IU/L), and myoglobin (488.7 *μ*g/L; normal range, 16.3–96.2 *μ*g/L). Antinuclear antibody and antiJo-1 antibody levels were negative. The nasopharyngeal carcinoma was treated first, and the dermatomyositis was treated second, because it was necessary to avoid the immunosuppression induced by prednisolone in case of neck dissections if there were residual tumors after chemoradiotherapy. On the second day of hospitalization, chemoradiotherapy was started. Radiotherapy of 66 Gy by Linac was administered to an area extending from the nasopharynx to the lower neck at a rate of 2 Gy/day. Simultaneously, weekly chemotherapy was initiated through an intravenous drip of 30 mg of nedaplatin and 20 mg of docetaxel repeated five times. The tumor size decreased rapidly within the first 2 weeks. However, the patient's dermatomyositis symptoms progressed; in particular, the CK level increased from 1490 IU/L to 2893 IU/L. Therefore, prednisolone at 60 mg per day was started. Two weeks later, the CK level decreased spontaneously to 332 IU/L. The patient's clinical course is shown in Figure 3. Five months after treatment was finished, positron emission tomography/computed tomography (PET-CT) showed no residual tumor so no neck dissections were carried out. Pre- and posttreatment PET-CT scans are shown in Figure 4.

## 3. Discussion

Dermatomyositis is well known to be associated with cancer; various studies have reported its incidence ranging from 7% to 34% [2]. The carcinoma types vary in each country. Hill et al. reported that ovarian, lung, gastric, colorectal, and pancreatic cancers were most strongly associated with dermatomyositis in several northern European countries [3], whereas Wong reported that nasopharyngeal carcinoma was as high as 75% in Hong Kong [4] and Ang et al. reported a rate of 50% in Singapore [5]. On the other hand, in Japan, gastric, lung, breast, and ovarian cancers were most associated with dermatomyositis (in order of frequency) [6].

Diagnostic criteria for dermatomyositis [7] are as follows: (1) progressive symmetric muscle weakness of the inferior and superior extremity girdle with or without dysphagia and with respiratory muscle involvement; (2) muscle biopsy confirming myositis; (3) increase in muscle enzyme serum levels; (4) electromyography abnormality indicating primary muscle damage; (5) characteristic skin lesions.

The presence of symptoms 1, 3, and 4 in Case 1 confirmed the diagnosis of dermatomyositis and the presence of symptoms 1, 2, 3, and 5 in Case 2 confirmed the diagnosis of dermatomyositis. Anti- Jo-1 antibody has a high diagnostic specificity, but its positive rate is only 30% of cases. Anti-Jo-1 level were negative in these two cases. Characteristically, both patients reported dysphagia as one of their first symptoms and the diagnoses of cancer and dermatomyositis were made almost simultaneously. In general, the risk of cancer is highest within the first year following diagnosis of dermatomyositis [3]. This makes the diagnosis and treatment of dermatomyositis difficult.

Dermatomyositis associated with cancer is generally more resistant to corticosteroid and cytotoxic therapies than idiopathic myositis. Dermatomyositis activity reflects the state of cancer and some reports have described improvement of dermatomyositis without immunosuppressive drugs after the resection of the cancers [8, 9]. Effective antitumor treatment may be accompanied by regression of the inflammation and yet it may result in progression in the stage of cancer [10].

In Case 1, it was apparent that the patient was experiencing acute dermatomyositis and acute interstitial pneumonia, which could be fatal. Therefore, treatment of the dermatomyositis with immunosuppressive drugs was given priority. In Case 2, multiple cervical lymphadenopathy was advanced, so it was necessary to avoid the immunosuppression induced by prednisolone in case of neck dissections if there were residual tumors after chemoradiotherapy. However, the patient's dermatomyositis symptoms progressed and oral prednisolone was started. Fortunately, complete remission was achieved at the end of chemoradiotherapy. If there had been residual tumor in the cervical region, surgery would not have been possible until the prednisolone dose had been tapered sufficiently and the opportunity to operate may have been missed.

In both cases, radiotherapy was used to treat the head and neck cancer because these two types of cancer have very good sensitivity to radiation. Teo et al. reported that acute confluent mucositis necessitating interruption of radiotherapy occurred in 8 of 9 nasopharyngeal carcinoma patients with dermatomyositis, and 2 of 9 experienced chronic radiation skin necrosis [11]. In addition, Hareyama et al. reported that two patients with nasopharyngeal carcinoma and dermatomyositis developed a severe skin reaction that resulted in a deep neck abscess after chemoradiotherapy [12]. Therefore, it is important to pay careful attention to skin and mucous reactions secondary to radiotherapy with or without chemotherapy.

In Case 2, concurrent chemoradiotherapy with nedaplatin and docetaxel was selected. Nedaplatin is easy to give in complicated cases because it improves renal dysfunction and does not need as much hydration as cisplatin. In addition, docetaxel does not induce strong skin and mucous damage compared to 5-fluorouracil or S-1. In both cases, no grade 3 or 4 adverse skin events occurred. Therefore, these two drug combinations appear to be good chemoradiotherapy regimens [13, 14].

In the present cases, the autoimmune mechanism that induced dermatomyositis was not clear and the absolute number of cancer cases with dermatomyositis is small, so further studies would be needed to establish the treatment.

## 4. Conclusion

We experienced two cases of head and neck cancer with dermatomyositis. Both cases were treated with chemoradiotherapy and complete remissions were achieved without grade 3 or 4 adverse events. In general, it is important to pay careful attention to skin and mucous reactions because sometimes treatments induce severe complications such as a deep neck abscess. 

In the present cases, the autoimmune mechanism that induced dermatomyositis was not clear and the absolute number of cancer cases with dermatomyositis is small, so further studies would be needed to establish the treatment.
